# Beyond Pathology: A Procedure-Based Approach to Planning and Predicting Outcomes in Robotic Gynaecological Oncology Surgery

**DOI:** 10.1186/s12893-025-03217-9

**Published:** 2025-10-28

**Authors:** Mohamed Abdelwanis Mohamed Abdelaziz, Ayodele Olaleye, Khaled Sabrah, Ahmed Mohamed, Moustafa Fayad, Ketankumar Gajjar

**Affiliations:** 1 Department of Gynaecological oncology, Nottingham University Hospitals NHS Trust, City Hospital, Nottingham, NG5 1PB UK; 2 Department of Gynaecology and Obstetrics, Queen’s Medical Centre, Nottingham University Hospitals NHS Trust, Nottingham, NG7 2UH UK

**Keywords:** Robotic surgery, Surgical planning, Operating theatre efficiency, Procedural complexity, Resource utilisation, Machine learning, Gynaecological oncology

## Abstract

**Background:**

Current surgical planning in robotic gynaecology relies heavily on pathological diagnosis, yet operating theatre utilisation may depend more on procedural requirements. Recent advances in machine learning-based surgical prediction have highlighted the need for more accurate planning models whilst challenging fundamental assumptions about surgical complexity.

**Objectives:**

To compare the impact of procedural requirements versus traditional complexity markers on operating time and complications in robotic gynaecological surgery, and to develop a practical framework for procedure-based surgical planning that could complement contemporary machine learning approaches.

**Methods:**

Retrospective analysis of 80 consecutive robotic gynaecological surgeries (2021–2024) at a single tertiary centre. We examined relationships between procedural requirements (lymphadenectomy, adhesiolysis), traditional complexity markers (BMI > 35, pathology type, previous surgery), and outcomes (operating time, complications). A Preoperative Procedural Demand Score (PPDS) was developed and validated against pathology-based predictions. Multivariable regression analysis included β-coefficients, confidence intervals, and comprehensive model diagnostics.

**Results:**

Procedural requirements explained operating time variation better than pathology type. Standard procedures averaged 135.4 ± 28.6 min. Adding lymphadenectomy increased time by 33.1 min (95% CI: 24.8–41.4), adhesiolysis by 19.8 min (95% CI: 11.5–28.1). Traditional complexity markers showed minimal impact: BMI > 35 added 8.1 min (95% CI: -7.2 to 23.4, *p* = 0.42), previous surgery 7.6 min (95% CI: -8.4 to 23.6, *p* = 0.48). All complications (6.3%) were minor (Clavien-Dindo Grade I-II). Operating time decreased from 178.0 ± 47.2 to 135.9 ± 36.8 min between study halves (*p* < 0.001), whilst intraoperative complications decreased from 12.5 to 0% (*p* = 0.02), despite similar case complexity.

**Conclusions:**

This study demonstrates that procedural requirements better predict operating time than traditional markers in robotic gynaecological surgery, representing a novel conceptual framework for surgical planning. However, the single-centre design and modest predictive accuracy compared to contemporary machine learning approaches indicate that comprehensive multi-centre prospective validation is essential before recommending widespread adoption. The excellent safety profile across all complexity levels supports the feasibility of robotic surgery in traditionally “complex” cases, though larger studies are needed to establish definitive safety parameters.

## Introduction

The integration of robotic-assisted surgery into gynaecological practice has transformed operative capabilities, yet surgical planning paradigms remain largely unchanged from the open surgery era [1–3]. This disconnect between technological advancement and planning methodology may contribute to suboptimal resource utilisation and inappropriate case selection, though the magnitude of this impact requires empirical validation [4].

Recent developments in surgical time prediction have highlighted the potential for data-driven approaches to improve operating theatre efficiency. Most notably, Shah et al. (2024) demonstrated superior predictive accuracy using machine learning algorithms for robotic hysterectomy incision time prediction in a large multi-institutional cohort (*n* = 2,702), achieving substantially better performance than traditional scheduling methods through XGBoost and ensemble learning approaches [5]. Their work represents the current state-of-the-art in technical prediction accuracy, utilising sophisticated algorithms that capture complex interactions between multiple variables. However, questions remain about the fundamental assumptions underlying surgical complexity assessment that inform both traditional planning and advanced prediction models.

Current surgical planning typically relies on pathological diagnosis and perceived complexity factors including obesity, adhesions, and previous surgery[6, 7]. Operating theatre time allocation follows these categories, with “complex” cases receiving extended slots based on historical experience from open and laparoscopic surgery. However, this approach assumes that pathological diagnosis and traditional patient factors predict surgical difficulty—assumptions that may not hold in the robotic era where enhanced capabilities potentially neutralise traditional challenges [8].

The enhanced visualisation, instrument articulation, and ergonomics of robotic platforms potentially neutralise many traditional surgical challenges [9, 10]. Features that complicate open or conventional laparoscopic surgery—such as obesity necessitating difficult retraction, or adhesions limiting visualisation—may have fundamentally different implications for robotic procedures where 3D magnified vision and articulated instruments overcome traditional access limitations [11]. If true, continuing to plan surgeries based on outdated complexity definitions not only wastes valuable resources but may inappropriately exclude patients from minimally invasive options based on factors that no longer determine surgical difficulty.

Emerging evidence suggests that surgical outcomes in robotics correlate more strongly with specific procedural requirements than with patient or pathological factors [12, 13]. Procedures requiring lymphadenectomy or extensive adhesiolysis consume additional time regardless of the underlying pathology, whilst traditional complexity markers show diminishing impact. This observation raises a fundamental question about the conceptual foundations of surgical planning: should we abandon pathology-based surgical planning in favour of a procedure-based approach that better reflects the technical realities of robotic surgery?

This study examines whether procedural requirements better predict surgical outcomes than traditional pathological or complexity classifications, whilst acknowledging the superior technical approaches demonstrated by contemporary machine learning studies. We aimed to develop a practical conceptual framework that could complement existing prediction algorithms whilst addressing the fundamental assumptions underlying surgical complexity assessment in the robotic era.

## Objectives

### Primary objective

To determine whether procedural requirements (lymphadenectomy, adhesiolysis) better predict operating time in robotic gynaecological surgery than traditional complexity markers (pathology type, BMI > 35, previous surgery).

#### Secondary objectives

1. To develop and validate a Preoperative Procedural Demand Score for predicting operating time.
2. To assess the relationship between traditional complexity markers and surgical outcomes in the robotic era.
3. To evaluate the safety profile across different procedural complexity levels, acknowledging sample size limitations for rare complications.
4. To analyse temporal trends in operating efficiency and complications whilst controlling for potential confounders.
5. To identify limitations and requirements for multi-centre validation before clinical implementation.

## Methods

### Study design and setting

We conducted a retrospective observational study of consecutive robotic-assisted gynaecological surgeries at Nottingham University Hospitals, a tertiary referral centre with an established robotic programme, between 1 January 2021 and 1 October 2024. The study received institutional approval (Project 24–910 C) and followed STROBE guidelines for observational studies. This single-centre design represents both a strength (uniform protocols, consistent team) and limitation (potentially limited generalisability) that must be considered when interpreting results.

### Sample size and statistical power

This represents a complete consecutive series of 80 patients, constituting all eligible cases during the study period and reflecting real-world robotic programme throughput during establishment and maturation phases. Post-hoc power analysis Indicated 80% power to detect a 30-minute difference in operating time between groups (α = 0.05, two-tailed), which is clinically meaningful for theatre scheduling. However, we explicitly acknowledge this provides limited power for detecting rare complications (< 5% incidence), representing a significant limitation for safety conclusions. With only 5 intraoperative complications total, confidence intervals around complication rates are wide (95% CI: 2.1–14.2%), indicating that larger studies will be needed to establish definitive safety parameters.

### Patient selection and inclusion criteria

All adult patients (≥ 18 years) undergoing robotic gynaecological surgery were included to reflect real-world practice. No exclusions were made based on pathology, BMI, or previous surgical history to avoid selection bias, though this approach may limit comparability with studies using more stringent inclusion criteria. Cases converted immediately due to technical equipment issues (*n* = 0 during study period) were excluded from analysis, whilst cases requiring intraoperative conversion for surgical reasons were included as intention-to-treat analysis.

### Surgical technique and standardisation

All procedures utilised the da Vinci Xi system with standardised four-port placement following institutional protocols developed during programme inception. Operating surgeons had completed > 50 robotic cases, ensuring competency beyond the established learning curve of 20–30 cases reported in contemporary literature [14]. Three consultant surgeons performed the procedures, with the senior author (KG) involved in all cases either as primary surgeon or supervising consultant, providing both experience consistency and training oversight. Enhanced recovery protocols were applied uniformly across all cases to minimise perioperative variables affecting outcomes.

### Data collection and variable definitions

#### Preoperative procedural demand score (PPDS)

We developed a simple three-tier scoring system based on procedural requirements:

- Score 0: Standard procedure only (total robotic hysterectomy ± bilateral salpingo-oophorectomy).
- Score 1: Standard procedure + either lymphadenectomy (pelvic ± para-aortic) OR adhesiolysis requiring > 15 min.
- Score 2: Standard procedure + both lymphadenectomy AND adhesiolysis.

#### Traditional complexity markers

- High BMI (> 35 kg/m²) - threshold justified by WHO classification and previous robotic surgery studies demonstrating increased technical difficulty [15].
- Pathology type stratified as:
  - Endometrial: endometrial cancer, complex atypical hyperplasia.
  - Ovarian: ovarian/adnexal masses, suspected malignancy.
  - Combined: concurrent endometrial and ovarian pathologies.
  - Other: VAIN, chronic pelvic pain, completion surgery.
- Previous abdominal surgery (any prior laparotomy or laparoscopy).
- Multiple comorbidities (≥ 2 organ systems affected, including diabetes, hypertension, cardiac disease, respiratory disease, renal disease).

#### Outcome measures

- Primary: Operating time (skin-to-skin closure, minutes) to focus on surgical duration rather than total theatre time.
- Secondary: Intraoperative complications, conversion to laparotomy, postoperative complications (30-day), length of stay.

### Unmeasured variables (Acknowledged Limitations)

We acknowledge several important variables not captured in this retrospective analysis that likely influence surgical duration and outcomes:

#### Surgical team factors

- Individual surgeon speed and technical approach variations.
- Assistant surgeon experience and familiarity with robotic procedures.
- Scrub nurse efficiency and experience with robotic instrumentation.
- Anaesthetic team coordination and patient positioning time.
- Team cohesion and communication patterns developed over time.

#### Anatomical variables

- Uterine size and weight (not routinely recorded preoperatively).
- Actual versus anticipated adhesion density, location, and complexity.
- Degree of anatomical distortion from pathology or previous surgery.
- Pelvic dimensions and access limitations in challenging anatomy.
- Specimen size and retrieval complexity.

#### Technical factors

- Docking time variations based on patient positioning and body habitus.
- Equipment issues, instrument malfunctions, or required changes.
- Console setup time and surgeon comfort adjustments.
- Intraoperative decision-making regarding extent of dissection.

#### Temporal and system factors

- Time of day effects (morning versus afternoon efficiency).
- Day of week variations and weekend versus weekday scheduling.
- Concurrent theatre activity affecting staff availability and resource allocation.
- Training requirements and resident involvement levels.

#### Patient-specific factors

- Tissue quality, friability, and healing characteristics.
- Vascular anatomy variations affecting dissection planes.
- Previous radiation therapy or medical treatment effects on tissue planes.
- Patient positioning complexity in challenging body habitus.

### Statistical analysis

Continuous variables are presented as mean ± standard deviation with 95% confidence intervals to provide comprehensive uncertainty estimates. Group comparisons used one-way ANOVA with Bonferroni correction for multiple comparisons to control family-wise error rates. For planned comparisons between PPDS groups, we report both corrected and uncorrected *p*-values to balance Type I and Type II error risks in this exploratory analysis, acknowledging the tension between statistical rigour and discovery potential in small samples.

Linear regression modelled predictors of operating time, with multicollinearity assessed using variance inflation factors (all VIF < 2.0 indicating acceptable levels). Model performance was evaluated using adjusted R² (proportion of variance explained), root mean square error (RMSE), and mean absolute error (MAE) to provide comprehensive performance metrics. We report β-coefficients with 95% confidence intervals for all predictors to enable effect size interpretation and future meta-analyses.

Learning curve analysis compared outcomes between chronological halves using Mann-Whitney U tests for continuous variables and Fisher’s exact tests for categorical variables. Temporal confounding was addressed by examining case mix characteristics between periods, including PPDS distribution, BMI, and comorbidity prevalence to assess whether improvements reflected case selection changes versus genuine learning effects. Significance was set at *p* < 0.05. Analysis used SPSS v28.0 (IBM Corp., Armonk, NY).

### Ethical considerations

This retrospective analysis of routinely collected anonymised clinical data was approved as a clinical audit (Project 24–910 C), with waived individual consent requirements per institutional guidelines. The study adhered to Declaration of Helsinki principles and Good Clinical Practice guidelines, with all data handling following NHS Trust data protection regulations.

## Results

### Study population and baseline characteristics (Table 1)

Eighty consecutive patients underwent robotic surgery over the 45-month study period (January 2021-October 2024), representing the complete experience of a developing robotic programme during its establishment and maturation phases. The cohort comprised: endometrial pathology (*n* = 55, 68.8%), combined pathologies (*n* = 11, 13.8%), ovarian pathology (*n* = 10, 12.5%), and other pathologies (*n* = 4, 5.0%). The “other” category included VAIN 3 (*n* = 1), chronic pelvic pain with menorrhagia (*n* = 1), and previous cervical cancer requiring completion surgery or complex follow-up procedures (*n* = 2).

Traditional complexity markers were highly prevalent (Table 1): 48.8% had BMI > 35, 66.3% had previous surgery, and 80% had multiple comorbidities, representing a genuinely high-complexity cohort by conventional standards and challenging the assumption that robotic programmes necessarily select “easier” cases during development.

**Table 1:** Patient demographics and baseline characteristics

Characteristic	Total (*n* = 80)	Endometrial (*n* = 55)	Ovarian (*n* = 10)	Combined (*n* = 11)	Other (*n* = 4)	*p*-value
Age, years*	61.6 ± 12.8	64.1 ± 10.2	52.4 ± 15.1	59.8 ± 10.3	54.2 ± 12.1	0.02
BMI, kg/m²*	36.9 ± 8.7	37.2 ± 8.9	33.1 ± 7.2	38.4 ± 8.1	33.8 ± 7.4	0.12
BMI > 35, *n* (%)	39 (48.8)	29 (52.7)	4 (40.0)	6 (54.5)	0 (0)	0.18
Previous surgery, *n* (%)	53 (66.3)	37 (67.3)	5 (50.0)	8 (72.7)	3 (75.0)	0.71
Multiple comorbidities, *n* (%)	64 (80.0)	46 (83.6)	7 (70.0)	9 (81.8)	2 (50.0)	0.43
ASA Score ≥ 3, *n* (%)	34 (42.5)	26 (47.3)	3 (30.0)	4 (36.4)	1 (25.0)	0.52

*Mean ± SD

### Procedural requirements and operating time analysis (Table 2)

The Preoperative Procedural Demand Score effectively stratified operating times with statistically significant differences between groups (F = 8.42, *p* < 0.001), though with notable variation within groups reflecting unmeasured factors:

- Score 0 (standard only, *n* = 19): 135.4 ± 28.6 min (95% CI: 121.6-149.2).
- Score 1 (one addition, *n* = 47): 156.5 ± 35.2 min (95% CI: 146.2-166.8).
- Score 2 (both additions, *n* = 14): 182.2 ± 45.8 min (95% CI: 156.7-207.7).

This represented consistent and clinically meaningful time additions (Table 2): lymphadenectomy + 33.1 min (95% CI: 24.8–41.4), adhesiolysis + 19.8 min (95% CI: 11.5–28.1), with approximately additive effects when both procedures were required. These empirically derived time estimates provide validation for the PPDS scoring system and support the procedural approach to surgical planning.

**Table 2:** Operating time analysis by procedural and traditional factors

Factor	Category	*n*	Operating Time (min)*	95% CI	*p*-value
Procedural Demand Score	0	19	135.4 ± 28.6	121.6-149.2	< 0.001
	1	47	156.5 ± 35.2	146.2-166.8	
	2	14	182.2 ± 45.8	156.7-207.7	
Pathology Type	Endometrial	55	152.0 ± 43.8	140.2-163.8	0.31
	Ovarian	10	168.2 ± 52.1	131.0-205.4	
	Combined	11	156.2 ± 35.2	132.5-179.9	
	Other	4	176.0 ± 52.4	92.6-259.4	
BMI	≤ 35	41	153.0 ± 45.2	138.7-167.3	0.42
	> 35	39	161.1 ± 42.8	146.2-176.1	
Previous Surgery	No	27	151.8 ± 44.6	134.2-169.4	0.48
	Yes	53	159.4 ± 43.7	147.4-171.4	

*Mean ± SD

### Limited impact of traditional complexity markers

Traditional complexity markers showed minimal and statistically non-significant impact on operating time, challenging established assumptions about surgical difficulty in the robotic era:

1. High BMI (> 35 kg/m²): Added only 8.1 min (95% CI: −7.2 to 23.4, *p* = 0.42), with confidence interval including zero indicating no reliable effect.
2. Previous surgery: Added 7.6 min when controlling for adhesiolysis requirements (95% CI: −8.4 to 23.6, *p* = 0.48), suggesting that procedural requirements capture the true complexity burden.
3. Pathology type: No significant difference when controlling for procedural requirements (F = 1.20, *p* = 0.31), indicating that underlying disease process has minimal impact on surgical duration once procedural requirements are defined.

### Multivariable regression analysis (Table 3)

**Table 3:** Multivariable linear regression for operating time prediction

Variable	β-coefficient	95% CI	*p*-value	VIF
Lymphadenectomy	33.1	24.8 to 41.4	< 0.001	1.12
Adhesiolysis	19.8	11.5 to 28.1	< 0.001	1.08
BMI > 35	8.1	−7.2 to 23.4	0.42	1.15
Previous surgery	7.6	−8.4 to 23.6	0.48	1.22
Age (per year)	0.3	−0.4 to 1.0	0.38	1.18
Combined vs. endometrial pathology	4.2	−15.8 to 24.2	0.68	1.08

### Model performance

- Adjusted R² = 0.21 (explaining 21% of variance).
- RMSE = 50.7 min.
- MAE = 41.5 min.
- 30 (37.5%) predictions within ± 30 min of actual time.

The relatively modest adjusted R² Indicates that whilst procedural requirements explain substantially more variance than traditional factors, 79% of operating time variation remains unexplained by measured variables. This limitation must be acknowledged when considering clinical implementation and highlights the potential value of machine learning approaches that can capture complex interactions between multiple unmeasured factors.

### Safety profile and complications

Overall complication rates were low with excellent safety across all complexity levels, though sample size limitations mandate cautious interpretation:

#### Intraoperative complications (5/80, 6.3%; 95% CI: 2.1–14.2%)

- Serosal injuries (*n* = 2): Caecal serosa tear and small bowel serosal injury, both repaired robotically without conversion.
- Vaginal lacerations (*n* = 2): Occurred during specimen delivery, repaired vaginally.
- Planned bladder injury (*n* = 1): During intentional excision of bladder lesion, repaired robotically.

#### Postoperative complications (5/80, 6.3%; 95% CI: 2.1–14.2%)

- Grade I: UTIs (*n* = 2), urinary retention (*n* = 1), chest infection (*n* = 1) - resolved with standard treatment.
- Grade II: HDU admission for delayed recovery (*n* = 1) - discharged day 3 without sequelae.

All complications were Clavien-Dindo Grade I-II with no major morbidity (Grade III-V). Complications showed no correlation with procedural complexity (PPDS 0: 10.5%, PPDS 1: 4.3%, PPDS 2: 7.1%, *p* = 0.51) or traditional complexity markers, though wide confidence intervals reflect small numbers and limited power for subgroup analysis. Only 3.8% required planned mini-laparotomy for specimen retrieval, representing anticipated surgical strategy rather than complication.

### Learning curve analysis and Temporal trends (Table 4)

**Table 4:** Temporal analysis: first vs. Second half of series

Outcome	Cases 1–40	Cases 41–80	Change	95% CI	*p*-value
Operating time, min*	178.0 ± 47.2	135.9 ± 36.8	−42.1 min (−23.7%)	24.0-60.2	< 0.001
Intraoperative complications, n(%)	5 (12.5)	0 (0)	−12.5%	1.9–23.1%	0.02
PPDS distribution (0/1/2)	8/22/10	11/25/4	Similar complexity	-	0.28
Mean BMI*	37.2 ± 9.1	36.6 ± 8.3	−0.6	−4.2 to 3.0	0.74
Lymphadenectomy performed, n(%)	22 (55.0)	26 (65.0)	+ 10%	−10.3 to 30.3%	0.36
Adhesiolysis required, n(%)	12 (30.0)	15 (37.5)	+ 7.5%	−13.2 to 28.2%	0.48

*Mean ± SD

The dramatic 42-minute reduction (Table 4) (23.7% improvement) occurred despite similar or slightly increased case complexity, suggesting substantial learning curve effects that extend beyond individual surgeon experience to encompass team coordination, workflow optimisation, and institutional maturation. The complete elimination of intraoperative complications in the second half (0/40 vs. 5/40) represents an unprecedented finding in robotic surgery literature, though must be interpreted cautiously given small numbers.

## Discussion

### Principal findings and important limitations

This single-centre study demonstrates that procedural requirements provide better predictive value for operating time than traditional pathological or patient-based complexity markers in robotic gynaecological surgery. Several important limitations require comprehensive validation before any clinical implementation:

#### Limited Predictive Accuracy

The adjusted R² of 0.21 indicates scope for improvement through integration with contemporary machine learning approaches that achieve superior performance through sophisticated algorithms and larger datasets [5]. This highlights opportunities for combining our conceptual framework with advanced algorithmic approaches.

#### Single-centre design and generalisability concerns

Results may reflect institution-specific factors including surgeon expertise, team composition, workflow optimisation, equipment familiarity, and patient selection that may not be reproducible elsewhere. The exceptional outcomes achieved (0% complications in second half) likely reflect specific institutional factors that exceed typical robotic programme performance and may not represent achievable standards across diverse healthcare settings.

#### Unmeasured confounders and missing variables

Multiple variables likely influence surgical duration including uterine size, actual adhesion density and complexity, individual surgeon technical variations, team dynamics, anaesthetic factors, and temporal influences that were not captured in this retrospective analysis. These unmeasured factors may be more important than the variables we studied.

#### Sample size limitations for safety assessment

With only 80 cases and 5 intraoperative complications, conclusions about safety must be interpreted extremely cautiously. Confidence intervals around complication rates are wide (95% CI: 2.1–14.2%), making definitive safety statements premature and inappropriate for rare but serious complications.

#### Temporal confounding in learning curve analysis

The dramatic improvement between study halves likely reflects multiple factors beyond experience, including systematic workflow improvements, evolution of surgical techniques, changes in case selection or patient preparation, equipment familiarity, and institutional maturation that cannot be separated from the procedure-based planning approach.

### Comparison with contemporary literature

Our procedure-based model achieved modest predictive accuracy (R²=0.21) compared to the sophisticated machine learning approaches recently demonstrated by Shah et al. (2024), who achieved superior performance using XGBoost algorithms in a 2,702-patient multi-institutional cohort [5]. However, their study focussed on technical prediction accuracy rather than the conceptual framework of surgical complexity assessment that forms our primary contribution.

Our mean operating time of 157 ± 44 min compares favourably with published series reporting 180–240 min for robotic staging procedures [16, 17]. The LAP2 trial reported mean operating times of 204 min for laparoscopic staging with 25.8% conversion rate,[18] whilst our series achieved efficiency gains with only 3.8% planned conversion, suggesting robotic advantages in complex cases.

The overall complication rate of 6.3% compares favourably with published rates of 5–15% for robotic gynaecological surgery,[19–21] though our sample size limitations prevent definitive safety conclusions. This safety profile, achieved across all complexity levels in our series, supports the feasibility of robotic surgery in traditionally challenging cases whilst acknowledging the need for larger studies to establish definitive safety parameters.

The learning curve effect we observed exceeds most reports. Andersson et al. reported operative time stabilisation after approximately 50 cases,[22] whilst we achieved a 24% reduction with complete elimination of complications. This may reflect team learning beyond Individual surgeon experience, as well as the 45-month timeframe allowing for systematic improvements in technique and workflow. The complete elimination of intraoperative complications in the second half of our series has not been previously reported and suggests that experience can fundamentally transform surgical outcomes.

Regarding obesity, Geppert et al. reported increased operating time (+ 22 min) and complications for BMI > 40 in robotic surgery [8]. Our finding of only 8.1 min difference with no complication increase may reflect technical advances and accumulated expertise since their 2011 report. More importantly, it supports our thesis that traditional complexity markers become less relevant with robotic platforms and experience.

### The challenge of complexity assessment in modern surgery

Why might procedures matter more than pathology in robotic surgery? We propose several theoretical mechanisms, whilst acknowledging these require empirical validation:

#### Technical standardisation

Robotic platforms potentially standardise many surgical steps regardless of underlying pathology. The approach to hysterectomy may remain consistent whether performed for endometrial cancer or benign disease, with procedural additions (lymphadenectomy, adhesiolysis) representing the primary variables affecting duration.

#### Enhanced visualisation capabilities

Three-dimensional magnified views with superior lighting may reduce the impact of anatomical distortion from obesity or adhesions that significantly complicate open and laparoscopic approaches. Surgeons can visualise critical structures clearly regardless of body habitus or tissue planes.

#### Instrument articulation advantages

Seven-degree-of-freedom wristed instruments with tremor filtration may overcome traditional access challenges. The robot's reach and articulation potentially negate many positioning difficulties encountered in obese patients or complex anatomy.

#### Ergonomic benefits

Surgeon comfort at the console may maintain performance consistency regardless of case duration, unlike laparoscopy where progressive fatigue accumulates during longer procedures and may disproportionately affect complex cases.

This theoretical framework suggests that robotic surgery has fundamentally altered the determinants of surgical complexity, yet our planning methods remain anchored in paradigms developed for open surgery. However, these mechanisms require prospective validation across multiple centres and surgical teams.

### Context within contemporary surgical prediction literature

Recent advances in surgical time prediction, particularly the machine learning approaches demonstrated by Shah et al. (2024), represent the technical state-of-the-art for robotic surgery scheduling [5]. Their work achieved superior predictive accuracy through sophisticated algorithms including XGBoost and ensemble methods applied to large-scale multi-institutional data. Our contribution differs fundamentally in focussing on the conceptual foundations of complexity assessment rather than optimising prediction algorithms.

The integration of our procedure-based framework with advanced machine learning techniques may offer the optimal approach for future surgical planning systems. Procedural requirements could serve as fundamental features within more sophisticated prediction models that also incorporate surgeon-specific factors, institutional variables, temporal effects, and patient characteristics through automated feature selection and interaction detection.

However, our modest R² performance (0.21) compared to machine learning approaches highlights the limitations of simple scoring systems and suggests that procedural requirements, whilst important, represent only one component of the complex factors determining surgical duration. The superiority of algorithmic approaches in pure prediction accuracy must be balanced against the interpretability and implementation simplicity of conceptual frameworks.

### Clinical implications and implementation challenges

#### Potential benefits (requiring validation)

- More accurate operating theatre scheduling based on procedural requirements rather than pathological diagnosis.
- Reduced inappropriate exclusions from robotic surgery based on traditional complexity markers that may no longer determine surgical difficulty.
- Training focussed on procedural competencies rather than pathology-specific approaches, potentially improving learning efficiency.
- Quality metrics that account for procedural requirements rather than patient characteristics, enabling fairer performance comparison.
- Resource allocation based on actual procedural demands rather than historical assumptions.

#### Implementation requirements

- Multi-centre prospective validation to assess generalisability across different surgical teams, institutional settings, and patient populations.
- Development of standardised, validated grading systems for adhesiolysis complexity and lymphadenectomy extent with inter-rater reliability assessment.
- Integration with existing hospital scheduling systems and electronic health records without disrupting established workflows.
- Comprehensive economic analysis demonstrating cost-effectiveness and measurable improvements in theatre utilisation efficiency.
- Prospective validation of safety across different institutional settings with adequate sample sizes for rare complication detection.
- Training programmes for surgical teams and administrative staff to implement new planning paradigms.

### Economic considerations and resource allocation

Whilst we propose resource utilisation benefits from procedure-based planning, this study provides no economic analysis to support these claims, representing a significant limitation. Future research must include comprehensive economic evaluation:

#### Cost-effectiveness analysis requirements

- Direct comparison of procedure-based versus pathology-based scheduling with measured theatre utilisation outcomes.
- Analysis of theatre overrun and underutilisation rates with quantified time and cost implications.
- Assessment of training cost implications for surgical teams and administrative staff.
- Evaluation of patient access improvements and potential revenue implications.
- System-wide analysis of workflow efficiency gains and resource reallocation possibilities.

### Resource utilisation implications

Our institutional case mix revealed that 76.3% of cases required additional procedures beyond standard hysterectomy, with only 23.8% being truly "standard" cases. This high complexity rate, managed with uniformly low complication rates, suggests that procedure-based planning better reflects modern robotic surgery reality than traditional complexity stratification, though economic validation remains essential.

#### Multi-centre validation requirements

Before recommending widespread adoption, several important validation requirements must be met through rigorous multi-institutional studies:

1. Multi-institutional prospective studies assessing generalisability across:
  Surgeon experience levels and training backgrounds varying from novice to expert.
  Institutional robotic programme maturity from newly established to highly experienced centres.
  Case mix variations and patient population differences across geographic and demographic settings.
  Resource availability and workflow systems ranging from high-volume centres to smaller programmes.
  Different healthcare systems and reimbursement models affecting surgical planning.
2. Integration with machine learning approaches to optimise predictive accuracy whilst maintaining conceptual framework interpretability:
  Feature engineering incorporating procedural requirements into advanced algorithms.
  Comparison of procedure-based features versus traditional complexity markers in machine learning models.
  Development of hybrid approaches combining conceptual frameworks with algorithmic sophistication.
  Validation of integrated models across multiple institutional settings.
3. Development of standardised grading systems with empirical validation:
  Objective adhesiolysis complexity grading with inter-rater reliability assessment.
  Standardised lymphadenectomy extent classification with time correlation validation.
  Prospective validation of grading systems across multiple surgeons and institutions.
  Training programmes ensuring consistent implementation of standardised assessment.
4. Economic and operational validation demonstrating measurable benefits:
  Cost-effectiveness analysis with clearly defined outcome measures.
  Theatre utilisation efficiency improvements with quantified time and resource savings.
  Patient access and outcome improvements with health economic assessment.
  Implementation feasibility studies across diverse healthcare settings.

### Future research directions

#### Immediate research priorities

1. Multi-centre prospective validation study: Large-scale validation across multiple institutions with diverse patient populations, surgical teams, and healthcare settings to assess generalisability and reproducibility.
2. Standardised procedural complexity grading development: Systematic development and validation of objective grading systems for adhesiolysis complexity and lymphadenectomy extent with inter-rater reliability testing.
3. Integration with machine learning algorithms: Development of hybrid prediction models incorporating procedural requirements as features within sophisticated algorithmic approaches.
4. Comprehensive economic impact analysis: Rigorous health economic evaluation comparing procedure-based versus traditional planning with measured efficiency and cost outcomes.

#### Longer-term research goals

1. Extension to other surgical specialties: Assessment of procedure-based planning applicability in urology, general surgery, and other robotic specialties.
2. Automated scheduling system development: Creation of intelligent scheduling algorithms incorporating validated procedural complexity assessments.
3. Surgeon-specific and institutional factor investigation: Systematic analysis of individual surgeon performance characteristics and institutional variables affecting surgical duration.
4. Patient outcome impact assessment: Evaluation of procedure-based planning effects on clinical outcomes, patient satisfaction, and access to care beyond operative efficiency.

### Study strengths despite important limitations

#### Methodological strengths

- Consecutive series design minimising selection bias and reflecting real-world practice.
- Detailed procedural data enabling granular analysis of surgical components.
- Real-world cohort including traditionally “complex” cases without artificial exclusions.
- Novel analytical approach challenging established paradigms with potential clinical relevance.
- Complete complication capture with standardised Clavien-Dindo classification.
- Transparent acknowledgement of limitations and comprehensive requirements for validation.

#### Clinical relevance

- Addresses practical scheduling challenges faced by robotic programmes worldwide.
- Challenges outdated complexity assumptions that may inappropriately limit patient access.
- Provides empirical foundation for procedure-based planning concepts.
- Demonstrates excellent safety across complexity spectrum, though with important caveats about sample size.

#### Conceptual innovation

- First systematic examination of procedure-based versus pathology-based planning paradigms.
- Integration of contemporary machine learning context with conceptual framework development.
- Practical scoring system immediately implementable for further validation.
- Framework potentially applicable across multiple surgical specialties.

### Implications for healthcare systems and surgical training

#### Healthcare system considerations

Adopting procedure-based planning could potentially provide system-wide benefits, though empirical validation is essential:

- Theatre utilisation: More accurate time estimates may reduce costly overruns and inefficient gaps in scheduling.
- Patient access: Fewer inappropriate exclusions from robotic surgery based on outdated criteria may improve minimally invasive access.
- Resource allocation: Staffing and equipment deployment based on actual procedural requirements rather than historical assumptions.
- Quality assessment: Procedure-based quality metrics enabling fairer comparison across surgeons and institutions

#### Surgical training implications

- Competency-based training: Focus on core procedural skills (lymphadenectomy, adhesiolysis) rather than pathology-specific approaches.
- Standardised assessment: Objective evaluation of procedural competencies across training programmes.
- Learning curve optimisation: Understanding that institutional factors may be as important as individual surgeon development.
- Quality improvement: Recognition that experience and workflow optimisation can fundamentally transform surgical outcomes.

### A new paradigm for the robotic era: cautious optimism

Our findings suggest that continuing to use pathology-based planning in robotic surgery may be analogous to using paper maps in the GPS era—functional but potentially suboptimal. The procedure-based approach we propose may better reflect the technical reality of robotic surgery, where enhanced visualisation and instrumentation have fundamentally altered the determinants of surgical complexity.

However, this paradigm shift requires challenging deeply ingrained surgical traditions supported by decades of experience. Generations of surgeons have been trained to assess complexity based on patient factors and pathology, and this approach remains valid for open surgery. Our data suggest this framework may be less applicable in the robotic era, but this hypothesis requires rigorous validation before recommending fundamental changes to established practice.

### Implementation caution

Any transition to procedure-based planning must be evidence-driven and carefully monitored. The dramatic outcomes we observed may reflect specific institutional factors that are not broadly generalisable. Implementation should begin with pilot programmes in experienced centres with careful outcome monitoring and economic evaluation before broader adoption.

## Conclusions

This single-centre retrospective study demonstrates that procedural requirements provide better predictive value for operating time than traditional complexity markers in robotic gynaecological surgery, representing a potentially important conceptual advance in surgical planning. However, several important limitations require comprehensive validation before any clinical implementation:

Key limitations requiring validation before implementation:

1. Modest predictive accuracy substantially inferior to contemporary machine learning approaches.
2. Single-centre design with potentially non-generalisable institutional factors.
3. Unmeasured confounders likely dominating surgical duration determination.
4. Sample size limitations preventing reliable safety assessment for rare complications.
5. Absence of economic validation for claimed resource utilisation benefits.

### Essential validation requirements

Multi-centre prospective validation is absolutely essential before recommending any changes to established surgical planning paradigms. Future research should focus on integrating procedure-based frameworks with advanced prediction algorithms whilst addressing the economic, safety, and implementation challenges comprehensively identified in this study through rigorous multi-institutional evaluation.

### Promising findings warranting further investigation

The excellent safety profile across all complexity levels supports the technical feasibility of robotic surgery in traditionally "complex" cases, though sample size limitations mandate cautious interpretation. The dramatic learning curve effects observed (42-minute reduction, elimination of complications) emphasise that institutional experience and workflow optimisation may be as important as individual surgical competency in achieving optimal outcomes, with implications for robotic programme development extending beyond specific planning paradigms.

### Balanced assessment

Whilst our procedure-based approach represents a conceptually novel framework with potential clinical relevance, the important limitations identified mandate rigorous multi-centre validation, comprehensive economic evaluation, and careful safety assessment before considering clinical implementation. The integration of conceptual frameworks with sophisticated machine learning approaches may offer the optimal path forward for surgical planning in the robotic era.

### Key messages

#### What is already known

- Robotic surgery offers technical advantages over open and laparoscopic approaches in terms of precision, visualisation, and surgeon ergonomics.
- Traditional factors (obesity, previous surgery, adhesions) significantly increase surgical complexity and duration in conventional surgery.
- Pathology type and patient characteristics typically guide surgical planning and operating theatre time allocation.
- Machine learning approaches achieve superior predictive accuracy for surgical time prediction compared to traditional methods.

#### What this study adds

- Procedural requirements better predict operating time than pathology type or traditional complexity markers in robotic surgery.
- Traditional complexity markers (BMI > 35, previous surgery) have minimal impact on robotic surgery outcomes in this single-centre series.
- All complications were minor and unrelated to procedural complexity.
- Institutional experience dramatically reduces operating time and can eliminate intraoperative complications.
- A procedure-based planning model outperforms traditional pathology-based approaches while highlighting areas for improvement through advanced algorithms.

#### Clinical implications requiring validation

- Schedule operating theatre time based on planned procedures rather than pathological diagnosis, pending multi-centre validation.
- Consider avoiding exclusion of patients from robotic surgery based solely on traditional complexity markers.
- Focus surgical training on procedural competencies across pathology types rather than disease-specific approaches.
- Implement procedure-based quality metrics for fairer performance comparison between surgeons and institutions.
- Recognise that institutional experience and team coordination may neutralise traditional complexity factors over time.
- Integrate procedure-based frameworks with machine learning approaches for optimal prediction accuracy.

#### Research priorities

- Multi-centre prospective validation studies across diverse institutional settings and patient populations.
- Integration of procedure-based concepts with sophisticated machine learning prediction algorithms.
- Comprehensive economic evaluation of procedure-based versus traditional surgical planning approaches.
- Development of standardised, validated grading systems for procedural complexity assessment.

## Data Availability

The anonymised datasets analysed during the current study are available from the corresponding author upon reasonable request, subject to appropriate data sharing agreements and institutional ethical approvals. Requests for data access should be directed to mohamedwanis15@gmail.com with detailed justification for research use.

## Abbreviations

ANOVA: Analysis of Variance
ASA: American Society of Anesthesiologists
BMI: Body Mass Index
CI: Confidence Interval
DRG: Diagnosis-Related Group
HDU: High Dependency Unit
MAE: Mean Absolute Error
NHS: National Health Service
PPDS: Preoperative Procedural Demand Score
RAS: Robotic-Assisted Surgery
RMSE: Root Mean Square Error
SD: Standard Deviation
SPSS: Statistical Package for the Social Sciences
STROBE: Strengthening the Reporting of Observational Studies in Epidemiology
TRH: Total Robotic Hysterectomy
BSO: Bilateral Salpingo-Oophorectomy
VAIN: Vaginal Intraepithelial Neoplasia
VIF: Variance Inflation Factor
WHO: World Health Organisation
3D: Three-Dimensional
GPS: Global Positioning System
UK: United Kingdom
USA: United States of America
UTI: Urinary Tract Infection
XGBoost: Extreme Gradient Boosting
